# Effects of cluster size on trypophobic discomfort in children aged 4–9 years

**DOI:** 10.1038/s41598-024-67002-z

**Published:** 2024-07-17

**Authors:** Tomoko Imura, Chiharu Suzuki, Mai Kasahara, Kyoshiro Sasaki, Yuki Yamada, Nobu Shirai

**Affiliations:** 1 Department of Psychology, Faculty of Integrated Arts and Social Sciences, Japan Women’s University, 2-8-1, Mejirodai, Bunkyo-ku, Tokyo, 112-8681 Japan; 2 Department of Psychology, Faculty of Integrated Arts and Social Sciences, Japan Women’s University, Tokyo, Japan; 3 Department of Psychology, Faculty of Humanities, Niigata University, Niigata, Japan; 4 Faculty of Informatics, Kansai University, Osaka, Japan; 5 Faculty of Arts and Science, Kyushu University, Fukuoka, Japan; 6 Department of Psychology, College of Contemporary Psychology, Rikkyo University, Saitama, Japan

**Keywords:** Trypophobia, Cluster size, Children, Remote experiment, Developmental biology, Psychology

## Abstract

It has been reported that strong discomfort associated with clusters of circles and holes (trypophobia), including lotus pod seeds, manifests in individuals as young as 4 or 5 years old. This study investigated how the size and number of circles within clusters affected discomfort levels in adults and in children aged 4–9 years. In Experiment 1, we confirmed that the remote experimental procedure could evoke discomfort when participants were presented with cluster images. The findings reveal that children as young as 4 or 5 years old consistently experienced discomfort when rating trypophobic images, even printed images rated in real time during video calls. In Experiment 2, we explored the impact of cluster size, considering both the size and number of circles, in a remote experiment. The results indicate that discomfort tended to increase with cluster size in both children and adults, with the effect becoming more pronounced with age.

## Introduction

Trypophobia is a common form of discomfort that is triggered by patterns of circles or holes, such as those found in lotus seed pods. Previous research suggests that approximately 16% of adults experience extreme discomfort with clusters^1^. Previous studies have explored individual variations in discomfort, considering factors such as personality traits and aversion to infection^2–5^. There has also been discussion about the influence of unique visual features in cluster images on discomfort^1,6,7^. However, most of these studies focused on adults. Consequently, our understanding of the development of discomfort with clusters in children is limited, and the prevalence of trypophobia in children is unknown.

It has been proposed that unpleasant emotions, including discomfort and fear, play a crucial role in avoiding danger, and that this process begins early during development^8,9^. For instance, fear of dangerous creatures, such as snakes, is common in both human infants and non-human primates^10,11^. Moreover, children as young as 4 or 5 years old have been shown to feel discomfort with clusters. When presented with images of clusters and toxic organisms, 4- and 5-year-olds tended not to favor clusters when asked to select their preferred images^12^. Recently, Suzuki et al. (2023)^13^ directly investigated childhood discomfort with cluster images, revealing that 4- to 9-year-olds, like adults, experienced higher levels of discomfort with photographs containing circular clusters of lotus seed pods or honeycombs compared to neutral images that showed a single hole. Images were rated on four dimensions (“discomfort,” “fear,” “itchiness,” and “like”). Although these studies indicate that discomfort with clusters can occur as early as preschool age, it remains unclear which specific characteristics of clusters children find discomforting.

It has been reported that specific spatial characteristics of cluster images can cause visual discomfort in adults^1,6,7^. It has also been demonstrated that visual features, including the number, size, overlap, and concavity of circular objects within clusters can also influence discomfort levels^4,14^. While previous developmental studies have used images that induce discomfort in adults as cluster stimuli, such as lotus seed pods^12,13^, few studies have directly examined discomfort with circular clusters in children^15^. In the present study, we generated cluster stimuli comprising black dots against a white background^4^ and examined how cluster size, determined by the size and number of circles, affected discomfort in children aged 4–9 years. Given the challenges posed by the COVID-19 pandemic, face-to-face experiments could not be conducted; instead, we conducted all experiments remotely. Initially, we replicated our previous face-to-face experiments^13^ using a new remote procedure (Experiment 1). Subsequently, we investigated the effect of cluster size on trypophobic discomfort in both children and adults through remote experiments (Experiment 2).

## Experiment 1. Discomfort related to trypophobic images: remote experiment

Experiment 1 aimed to determine whether the results of a face-to-face experiment reported by Suzuki et al. (2023)^13^ could be replicated through a remote experiment analyzing children’s discomfort with trypophobic images.

### Participants

Experiment 1 included 60 children aged 4–9 years and 20 adults. Table 1 shows the age and sex distribution of the participants in each age group. Eleven additional children were initially involved but were excluded from the final sample. Six of these participants could not complete the experiment because of discomfort (n = 3), video-recording issues (n = 2), or an unstable Internet connection (n = 1). Furthermore, children who did not rate all items (n = 4) and those whose responses were inconsistent (n = 1) were excluded from the final analysis.

**Table 1:** Age and sex distribution of each age group in Experiment 1.

Age group	Age in years		Sex		
	M	SD	Males	Females	Total
4–5 years old	5.07	0.62	10	10	20
6–7 years old	7.06	0.56	11	9	20
8–9 years old	8.82	0.67	10	10	20
Adults	22.31	3.25	10	10	20

Participants were recruited via flyers distributed at public health centers in Niigata City, Japan, and through the website of the research laboratory of the Department of Psychology, Niigata University. All participants received a ¥1,000 bookstore gift card as a reward after the experiment.

The sample size (n = 20 per age group) was determined as in the study by Suzuki et al. (2023)^13^. Because there were few similar previous studies, it was difficult to estimate the required sample size. Thus, in accordance with the recommendation of Simmons et al.^16^, an heuristic criterion was used (≥ 20 participants per group) to minimize the risk of type I error.

### Ethics statement

This study was conducted in accordance with the Declaration of Helsinki and was approved by the Ethics Committee for Human Research at Niigata University, Japan (approval no. 2017-0359 v4). Written informed consent for study participation was obtained prior to the experiment. Children were asked to provide written informed assent if they were able to sign their own name; written informed consent was provided by each child's parents. Written informed consent was also obtained from all adult participants. Before the informed consent process, participants were shown thumbnail images of the stimuli. They were then informed that if they experienced significant discomfort when viewing the images, they could withdraw from the study; this could be done at any time (i.e., before or after the experiment began).

### Stimuli

In total, 41 images were used in the study, consisting of 40 test stimuli and 1 practice stimulus. The test stimuli comprised 20 cluster images (including lotus seed pods, honeycombs, etc.) and 20 neutral images having the common feature of a single hole. These images were originally provided by Le et al. (2015)^4^ and were used in a developmental study by Suzuki et al. (2023)^13^. An additional sunflower image was used as the practice stimulus.

To ensure consistency in stimulus size and image quality across devices, all stimuli were printed on A4 size glossy photo paper (BKS170-A4 premium photo-gloss paper; Pictorico) using an inkjet printer (DCP-J963N; Brother). Ten test stimuli were prepared for each participant, five each randomly selected from the cluster- and neutral-image sets. These 10 stimuli, together with 1 practice stimulus, were compiled into a booklet. Each page of the booklet featured a single image (11.5 × 11.5 cm), a four-point Likert scale, and a gray background. To enable tracking, each image and the Likert scale were numbered, allowing the experimenter to identify the specific page the participant was viewing during the remote experiment. The order of presentation of the 10 test images and the 4 rating items was randomized across participants.

Each participant received their booklet before the experiment and returned it to the experimenter afterwards via a parcel delivery service. Together with the booklet, instruction papers, consent forms, and a smartphone/tablet stand (see Procedure for details) were also sent to the participants.

The edges of the front cover and the pages of each booklet were firmly held together using two binder clips, which prevented booklet opening before the start of the experiment. Through an instruction leaflet shipped together with the booklet, parents were instructed to not open the booklet until the beginning of the experimental session. Additionally, a cautionary message (“Do not open this booklet before starting the experiment”) was printed in large, prominent letters on the front cover of the booklet.

### Procedure

Each experiment took place in the participants’ homes, with the experimenter using video call applications, such as Zoom (Zoom Video Communications, Inc.) or Facetime (Apple), on a laptop computer (MacBook Pro, 15.4 inches; Apple) to provide instructions remotely. The participants connected using their own camera-equipped devices, including smartphones, tablets, and personal computers. To ensure proper camera framing, participants using smartphones or tablets were provided with a stand in advance. For child participants, parents were instructed not to place any items on their desks other than the booklets and electronic devices used for the video calls. Parents were also advised to remain behind the participants to avoid disruption. Other family members were requested not to enter the room where the experiment was being conducted. The participants’ and parents’ responses were recorded through the video call application with their consent.

At the beginning of the experiment, the investigator confirmed that the booklet had not been opened, and instructed the parent to remove the binder clips from the booklet in front of the camera, during an online video call. The experiment began with participants opening the booklet and following the instructions provided by the experimenter. They were asked to rate each image on four dimensions (“disgust,” “fear,” “itchiness,” and “like” [reverse-scored]). The four dimensions were taken from the Japanese version of the Trypophobia Questionnaire^17^, which was developed to measure sensitivity to trypophobia and can be easily understood by young children. The adjectives were written in hiragana, appearing at both ends of the Likert scale. The same Likert scale was used for adults. Participants verbally expressed their response (“not at all”, “not much”, “a little”, or “very much”). The experimenter recorded the responses. For child participants who had difficulty reading, the experimenter provided verbal support. For example, regarding the “like” rating, the experimenter asked the participant, “Do you like this picture or not?”; if the participant answered “yes”, they were then asked, “How much do you like it?” (e.g., “a little” or “very much”). To avoid confusion, we did not include a "neither" option.

Single images were rated on the four dimensions in each trial. The experiment consisted of 1 practice trial and 10 test trials, for a total of 11 trials. The standard adult set (Le et al., 2015) includes 20 trypophobic images and 20 neutral images. However, considering the potential for fatigue and distraction in children evaluating many images, we randomly selected five images from each condition for evaluation in this experiment. The participants were allowed to take breaks or pause the experiment at any time.

Adult participants and the parents of the children were instructed to turn the pages of the booklet and verify the image number and rating item number. Parents were advised not to look at the images in the booklet or engage with the children while they evaluated the images.

### Data analyses

Before conducting data analysis, we reviewed videos of the experiment and excluded invalid trials. The criteria for trial exclusion were parental intervention, uncertain ratings, and inconsistent responses. Based on these criteria, approximately 2.3% of all responses of the 4- and 5-year-olds were excluded. Subsequently, we computed the average rating for each image, assigning a value of 1 for “not at all”, 2 for “not much”, 3 for “a little”, and 4 for “very much.” For statistical tests, we set the alpha level at 0.05. A mixed-design analysis of variance (ANOVA) of the ratings was performed, including age group (4–5 years, 6–7 years, 8–9 years, adult) as a between-group factor and image category (neutral, trypophobic) as a within-group factor. In cases of a significant interaction, we subsequently tested for simple main effects. In cases of a significant simple main effect of age group, we performed the Holm multiple comparison test. We calculated *F*-values, *t*-values, *p*-values, and effect sizes (i.e., η^2^ and Cohen’s *d*).

## Results and discussion

**Figure 1:**
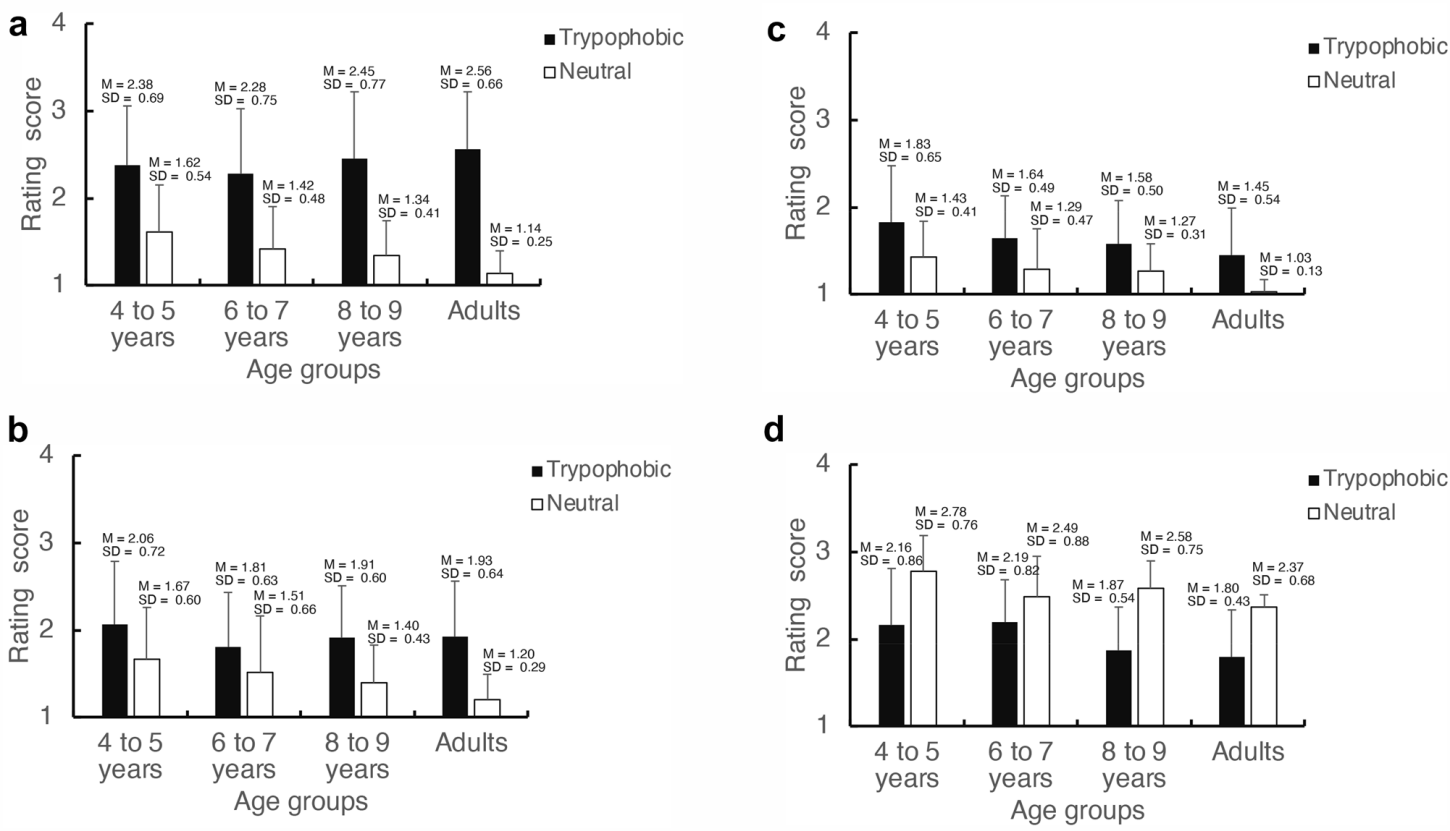
Mean ratings and standard deviations for “disgust” (**a**), “fear” (**b**), “itchiness” (**c**), and “like” (**d**) across age groups in Experiment 1. Black bars represent the results for trypophobic images, whereas white bars represent the results for neutral images. Error bars represent the standard deviation of the mean.

Figure 1a–d presents the average ratings and standard deviations for the four dimensions of trypophobic and neutral images according to age group (4–5 years, 6–7 years, 8–9 years, adult) (Table 2).

**Table 2:** Results of mixed-design two-way ANOVA including age group (4–5 years, 6–7 years, 8–9 years, adults) and image type (neutral, trypophobic): Experiment 1.

	(a) Disgust	(b) Fear	(c) Itchiness	(d) Like
Age group (4–5 years, 6–7 years, 8–9 years, adults)	*n.s*	*n.s*	4–5 years > adults*	*n.s*
Image type (neutral, trypophobic)	Trypophobic > neutral***	Trypophobic > neutral***	Trypophobic > neutral***	Trypophobic < neutral***
Age group × image type	4–5 years: Trypophobic > neutral*** 6–7 years: Trypophobic > neutral*** 8–9 years: Trypophobic > neutral*** adults: Trypophobic > neutral***	*n.s*	*n.s*	*n.s*

*n.s*., not significant.

*p < .05; **p < .01; ***p < .001.

(a) Disgust.

As shown in Fig. 1a, disgust ratings were significantly higher for trypophobic images than for neutral images across all age groups. Two-way ANOVA including age group and image category revealed that the main effect of age group was not significant (*F*[3, 76] = 0.44, *p* = 0.728, η^2^ = 0.02). However, the main effect of image category was highly significant (*F*[1, 76] = 158.38, *p* < 0.001, η^2^ = 0.67), and the interaction of age group and image category was also significant (*F*[3, 76] = 3.19, *p* = 0.028, η^2^ = 0.11). Multiple comparisons revealed significant differences between trypophobic and neutral images in all age groups (4–5-year-olds: *t*[76] = 4.609, *p* < 0.001, *d* = 1.713; 6–7-year-olds: *t*[76] = 5.216, *p* < 0.001, *d* = 1.425; 8–9-year-olds: *t*[76] = 6.732, *p* < 0.001, *d* = 1.839; adults: *t*[76] = 8.612, *p* < 0.001, *d* = 2.352); the ratings of all age groups were significantly higher for trypophobic images than for neutral images.

(b) Fear.

Ratings for “fear” were significantly higher for trypophobic images than for neutral images in all age groups (Fig. 1b). Two-way ANOVA including age group and image category indicated that only the main effect of image category was significant (*F*[1, 76] = 31.62, *p* < 0.001, η^2^ = 0.15). There was no main effect of age group (*F*[3, 76] = 1.64, *p* = 0.186, η^2^ = 0.03) and no significant age group × image category interaction (*F*[3, 76] = 1.17, *p* = 0.33, η^2^ = 0.02).

(c) Itchiness.

Ratings for “itchiness” were higher for trypophobic images than for neutral images in all age groups (Fig. 1c). Two-way ANOVA including age group and image category showed significant main effects of image category (*F*[1, 76] = 40.62, *p* < 0.001, η^2^ = 0.13) and age group (*F*[3, 76] = 3.55, *p* = 0.018, η^2^ = 0.08). Multiple comparisons revealed that the ratings of 4–5-year-olds were higher than those of adults (*t*[76] = 3.246, *p* = 0.010, *d* = 1.006). However, the age group × image category interaction was not significant (*F*[3, 76] = 0.18, *p* = 0.91, η^2^ = 0.001).

(d) Like.

Ratings for the reverse-scored “like” dimension were higher for neutral images than for trypophobic images across all age groups (Fig. 1d). A two-way ANOVA including age group and image category revealed a significant main effect of image category (*F*[1, 76] = 78.70, *p* < 0.001, η^2^ = 0.12). There was no significant main effect of age group (*F*[3, 76] = 1.18, *p* = 0.322, η^2^ = 0.03) and no significant age group × image category interaction (*F*[3, 76] = 2.02, *p* = 0.118, η^2^ = 0.009).

In summary, children in all age groups exhibited greater discomfort with trypophobic images compared to neutral images, with variations observed across rating items. These results imply that children begin to experience discomfort with cluster images around the age of 4 or 5 years. These findings also indicate that the remote experimental procedure successfully replicated Suzuki et al. (2023)^13^. In the study reported by Suzuki et al. (2023), the ratings for several items differed according to age. For example, “disgust” ratings for trypophobic images tended to increase with age, whereas “fear” ratings for neutral images tended to decrease with age. Additionally, "like" ratings were higher for 4–5-year-olds and adults than for the other age groups. In contrast, there were no significant differences in “disgust”, “fear”, or “like” ratings among the age groups, although there was a trend toward higher values for “itchiness” ratings in the 4–5-year-old group than in the adult group. Despite the differences in results between the present and previous studies, discomfort with trypophobic images occurred in all age groups in our study.

## Experiment 2. Effects of cluster size on trypophobic discomfort in children

In Experiment 1, children from 4–5 years of age onward showed discomfort when viewing images containing clusters. The results confirmed that remote experiments are useful for investigating trypophobic discomfort in both children and adults. Experiment 2, another remote experiment, was designed to address the main aim of the study. We examined children's discomfort when viewing clusters of circles, then clarified the relationship between the visual characteristics of clusters and the degree of discomfort. Previous studies with adults have shown that the number, size, and three-dimensional shape of circles can influence the level of discomfort. For example, Le et al. (2015)^4^ defined cluster size based on the number and size of elements, then manipulated these parameters using black circles on a white background as elements. They found that, as the element number increased and element size decreased (resulting in a larger cluster size), discomfort in the response to cluster stimuli increased. Although no developmental studies have manipulated circle number or size, it has been reported that 3–4-year-old children show greater discomfort when viewing clusters of circles than when viewing clusters of squares or equilateral triangles^15^. Therefore, in this study, children aged 4–9 years were asked to rate the clusters of circles according to the dimensions of disgust, fear, itchiness, and preference when the element number and size were manipulated.

### Participants

Experiment 2 included 60 children aged 4–9 years and 20 adults. The age and sex distribution of the participants in each age group is presented in Table 3. Two additional children were initially involved in the experiment but were excluded: one 6-year-old girl due to the experimenter’s inability to record a video of the experiment, and one 8-year-old boy because of sibling interference. All participants were newly recruited; none of them had participated in Experiment 1. Sample size determination and informed consent procedures were identical to those in Experiment 1.

**Table 3:** Age and sex distribution for each age group in Experiment 2.

Age group	Age in years		Sex		
	M	SD	Males	Females	Total
4–5 years old	5.07	0.62	10	10	20
6–7 years old	7.06	0.56	11	9	20
8–9 years old	8.82	0.67	10	10	20
Adults	22.31	3.25	10	10	20

### Ethics statement

This study was conducted in accordance with the Declaration of Helsinki and was approved by the Ethics Committee for Human Research at Niigata University, Japan (approval no. 2017-0359v4). As in Experiment 1, written informed consent for study participation was obtained from all child participants or their parents prior to the experiment, as well as from all adult participants. Thumbnail images of the stimuli were shown to the participants (and the parents of the children) before they provided informed consent or assent, as described above. Participants were then informed that they could withdraw from the experiment at any time if they felt uncomfortable when viewing the images.

### Stimuli

**Figure 2:**
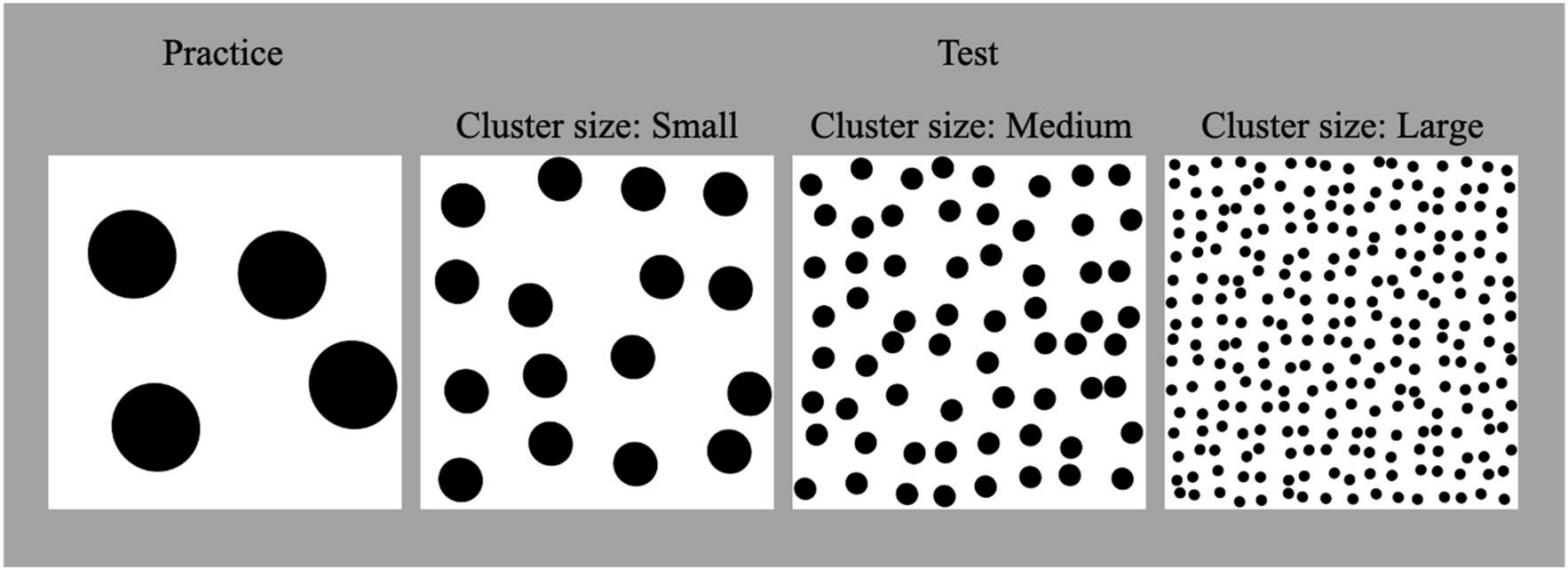
Example cluster stimuli used in the practice trial, and the three cluster conditions for test trials in Experiment 2.

In total, 16 stimuli were used in Experiment 2, comprising 15 test stimuli and 1 practice stimulus. To investigate the impact of the size and number of cluster elements, we created patterns comprising black dots randomly arranged against a white background (Fig. 2). Table 4 details the numbers and diameters of the dots for the practice and test stimuli under three cluster-size conditions. As the size of the clusters increased, the number of dots increased by a factor of 4, and the diameter of the dots decreased by a factor of 1/2. To mitigate the influence of dot placement, we used five different stimulus arrangements for each cluster-size condition.

**Table 4:** Number and size of cluster stimulus elements for Experiment 2.

Trial type	Cluster size	Number of circles	Diameter of circles (pixel)
Practice test	–	4	128
	Small	16	64
	Medium	64	32
	Large	256	16

All printed stimuli were generated following procedures similar to Experiment 1. Three test stimuli were prepared for each participant. Within each cluster-size condition, one of five stimulus arrangements was randomly selected. One practice stimulus and three test stimuli were compiled into one booklet. Each page of the booklet featured one stimulus (11.5 cm × 11.5 cm) against a gray background, together with a four-point Likert scale (19.6 cm). For adult participants, the order of presentation of the three test images and the four rating items was randomized across participants. Child participants responded to one practice stimulus and three different test stimuli for each rating item. Rating items were kept consistent to alleviate the reading burden on children. The order of the four rating items was randomized for each child participant.

The booklets were sent to the participants before the experiment and returned to the experimenter afterward through a parcel delivery service. Instruction papers, consent forms, and a smartphone/tablet stand (see Procedure for details) were also provided to the participants.

### Procedure

As in Experiment 1, the experiment was conducted at the participants' homes using a video-call application and a booklet containing the stimuli and Likert scales. For child participants who had difficulty reading, the experimenter provided verbal support.

In contrast to Experiment 1, participants rated one image in a single dimension during each trial; this process was repeated for three different cluster sizes. All four dimensions were covered in this manner during the experiment. The experiment consisted of 4 practice trials and 12 test trials, for a total of 16 trials. Participants could take a break or pause the experiment at any time; the order of presentation of the three cluster sizes and four rating dimensions was counter-balanced across participants. The instructions given to parents were similar to those in Experiment 1.

### Data analyses

We calculated the average rating for each image by assigning a score of 1 for “not at all,” 2 for “not much,” 3 for “a little,” and 4 for “very much.” A mixed-design ANOVA with age group as a between-group factor and cluster size as a within-group factor was performed for each rating item.

## Results and discussion

**Figure 3:**
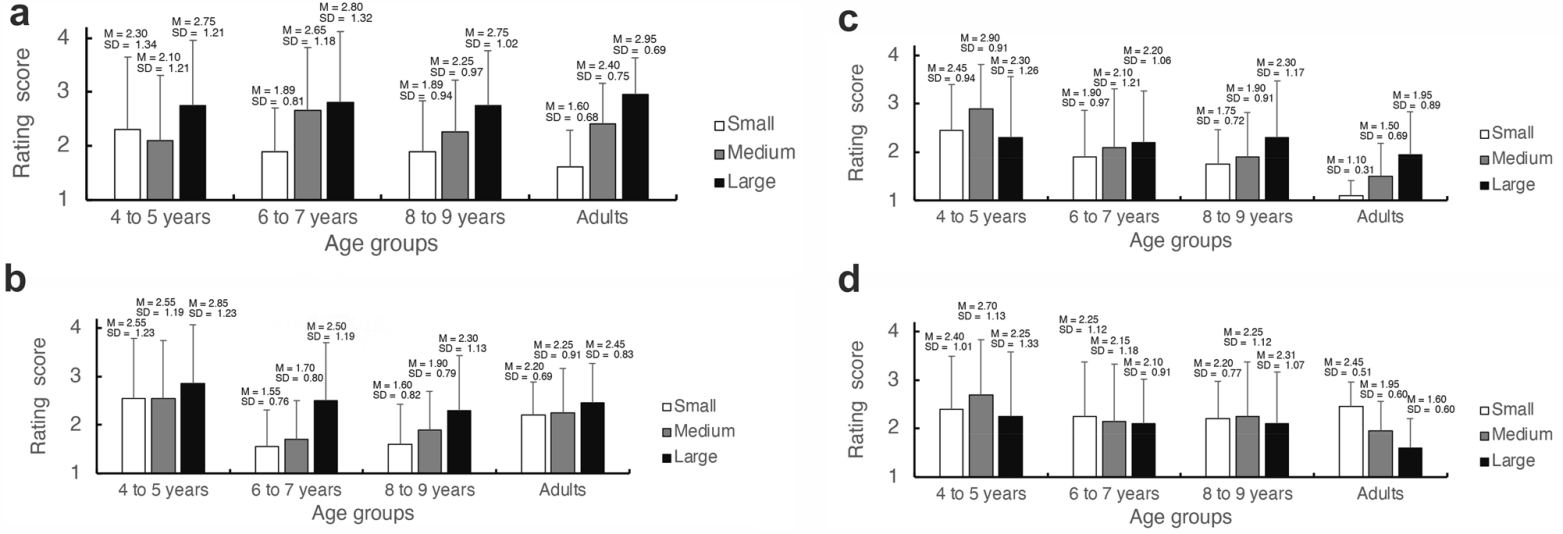
Mean ratings and standard deviations for “disgust” (**a**), “fear” (**b**), “itchiness” (**c**), and “like” (**d**) across the age groups in Experiment 2. Black, gray, and white bars represent the results for the large, medium, and small cluster sizes, respectively. Error bars represent the mean ± standard deviation of the mean.

The Fig. 3a–d depicts the average ratings and standard deviations for the four rating items (disgust, fear, itchiness, and like) in each cluster-size (small, medium, large) condition across the four age groups (Table 5).

**Table 5:** Results of mixed-design two-way ANOVA including age group (4–5 years, 6–7 years, 8–9 years, adults) and cluster size (small, medium, large): Experiment 2.

	(a) Disgust	(b) Fear	(c) Itchiness	(d) Like
Age group (4–5 years, 6–7 years, 8–9 years, adults)	*n.s*	4–5 years > 6–7 years* 4–5 years > 8–9 years*	4–5 years > adults*	*n.s*
Cluster size (small, medium, large)	Large > medium* Large > small*** Medium > small**	Large > medium** Large > small*** Medium > small**	Large > small** Medium > small***	*
Age group × image type	4–5 years: large > medium* 6–7 years: large > small**, medium > small* 8–9 years: large > small***	*n.s*	Adults: large > small** Medium > small*	*n.s*

*n.s*., not significant.

*p < .05; **p < .01; ***p < .001.

(a) Disgust.

The ratings for disgust increased with cluster size in all age groups (Fig. 3a). Two-way ANOVA including age group and cluster size revealed that the main effect of age group was not significant (*F*[3, 76] = 0.22, *p* = 0.882, η^2^ = 0.009). However, the main effect of cluster size was significant (*F*[2, 152] = 26.75, *p* < 0.001, η^2^ = 0.260). Multiple comparisons (Holm’s method) indicated significant differences among the cluster-size conditions: the ratings for “cluster size 256” were significantly higher than those for “cluster size 64” (*t*[76] = 3.699, *p* = 0.001, *d* = 0.432) and “cluster size 16” (*t*[76] = 7.905, *p* < 0.001, *d* = 0.835). The ratings for cluster size 64 were also significantly higher than those for cluster size 16 (*t*[76] = 3.358, *p* = 0.001, *d* = 0.402). This result indicates that discomfort becomes more pronounced as cluster size increases.

Moreover, the interaction between age group and cluster size was significant (*F*[6, 152] = 2.203, *p* = 0.046, η^2^ = 0.080), and there was a simple main effect of cluster size for all age groups (4- and 5-year-olds: *F*[2, 152] = 3.87, *p* = 0.023, η^2^ = 0.09; 6- and 7-year-olds: *F*[2, 152] = 6.31, *p* = 0.002, η^2^ = 0.15; 8- and 9-year-olds: *F*[2, 152] = 7.10, *p* = 0.001, η^2^ = 0.17; adults: *F*[2, 152] = 16.08, *p* < 0.001, η^2^ = 0.39). In adults, there were significant differences among the cluster-size conditions (Holm’s method), with the ratings for the large cluster size being significantly higher than those for the medium (*t*[76] = 2.199, *p* = 0.031, *d* = 0.506) and small (*t*[76] = 6.098, *p* < 0.001, *d* = 1.269) cluster sizes, and the ratings for the medium cluster size being significantly higher than those for the small cluster size (*t*[76] = 3.256, *p* = 0.003, *d* = 0.768). In children, there was a trend toward increasing discomfort with increasing cluster size. Among 4- and 5-year-olds, the ratings for the large cluster size were significantly higher than those for the medium cluster size (*t*[76] = 2.599, *p* = 0.034, *d* = 0.598), while in 6- and 7-year-olds, the ratings for the large and medium cluster sizes were significantly higher than those for the small cluster size (large vs. small: *t*[76] = 3.614, *p* = 0.002, *d* = 0.752; medium vs. small: *t*[76] = 2.646, *p* = 0.020, *d* = 0.624). In 8- and 9-year-olds, the ratings for the large cluster size were significantly higher than those for the small cluster size (*t*[76] = 4.065, *p* < 0.001, *d* = 1.269). This indicates that discomfort increased with cluster size in adults.

(b) Fear.

The ratings for fear also increased with cluster size in all age groups (Fig. 3b). Two-way ANOVA including age group and cluster size revealed significant main effects of both factors (age: *F*[3, 76] = 4.15, *p* = 0.009, η^2^ = 0.141; and cluster size: *F*[2, 152] = 17.92, *p* < 0.001, η^2^ = 0.191, respectively). However, the age group × image category interaction was not significant (*F*[6, 152] = 1.51, *p* = 0.18, η^2^ = 0.056). Multiple comparisons (Holm’s method) demonstrated significant differences among all cluster-size conditions, with the ratings for the large cluster size being significantly higher than those for the medium (*t*[76] = 3.767, *p* = 0.001, *d* = 0.413) and small (*t*[76] = 5.242, *p* < 0.001, *d* = 0.716) cluster sizes, and the ratings for the medium cluster size being significantly higher than those for the small cluster size (*t*[76] = 2.674, *p* = 0.009, *d* = 0.325). The ratings for 4- and 5-year-olds were higher than those for the other age groups (6- and 7-year-olds: *t*[76] = 3.059, *p* = 0.018, *d* = 0.948; 8- and 9-year-olds: *t*[76] = 2.989, *p* = 0.019, *d* = 0.927), implying that fear intensified as cluster size increased in all age groups, with 4- and 5-year-olds exhibiting stronger fear responses compared to the other age groups.

(c) Itchiness.

The ratings for itchiness also increased with cluster size in all age groups (Fig. 3c). Two-way ANOVA including age group and cluster size indicated significant main effects for both factors (age: *F*[3, 76] = 6.10, *p* < 0.001, η^2^ = 0.194; and cluster size: *F*[2, 152] = 6.85, *p* = 0.001, η^2^ = 0.083, respectively). Multiple comparisons (Holm’s method) revealed that 4–5-year-olds had higher itchiness ratings compared to adults (*t*[76] = 4.264, *p* < 0.001, *d* = 1.322). Moreover, the ratings were significantly higher for the large and medium cluster sizes than for the small cluster size (large vs. small: *t*[76] = 3.253, *p* = 0.003, *d* = 0.404; medium vs. small: *t*[76] = 3.468, *p* = 0.003, *d* = 0.344). Moreover, the age group × image category interaction was also significant (*F*[3, 76] = 6.85, *p* < 0.01, η^2^ = 0.03). There was a simple main effect of cluster size for all age groups, except for 6- and 7-year-olds (4- and 5-year-olds: *F*[2, 152] = 4.04, *p* = 0.020, η^2^ = 0.07; 8- and 9-year-olds: *F*[2, 152] = 3.35, *p* = 0.038, η^2^ = 0.06; adults: *F*[2, 152] = 7.49, *p* < 0.001, η^2^ = 0.13). Multiple comparisons revealed that the ratings for the large and medium cluster sizes were significantly higher than those for the small cluster size in adults (large vs. small: *t*[76] = 3.568, *p* = 0.001, *d* = 0.872; medium vs. small: *t*[76] = 2.312, *p* = 0.047, *d* = 0.451). This indicated that itching tended to increase with cluster size in all age groups, especially in adults. As with the ratings for fear, 4- and 5-year-olds exhibited a stronger overall itching response than the other age groups.

(d) Like.

In contrast to the other rating items, the ratings for the reverse-scored like item decreased with cluster size in all age groups (Fig. 3d). Two-way ANOVA including age group and cluster size showed a significant main effect of cluster size (*F*[2, 152] = 3.72, *p* = 0.028, η^2^ = 0.047), but there was no main effect of age group (*F*[3, 76] = 1.19, *p* = 0.320, η^2^ = 0.045) and no significant age group × image category interaction (*F*[6, 152] = 1.56, *p* = 0.167, η^2^ = 0.058). Multiple comparisons (Holm’s method) revealed no significant differences according to age group in the ratings for any cluster size.

In summary, there was a trend toward an increase in disgust, fear, and itchiness ratings with larger cluster sizes, while liking responses tended to decrease with larger cluster sizes. Although this pattern was also observed in children, it was more pronounced in adults. These results indicate that visual features causing discomfort in adults^4^ also cause discomfort in children. However, considering that other visual factors, such as element shape, may influence the degree of discomfort in children^15^, the effects of multiple visual features should be examined in the future.

## General discussion

In Experiment 1, we replicated the discomfort experienced by 4- to 9-year-olds in relation to trypophobic images in a remote setting. Discomfort with trypophobic images was consistently observed in 4- and 5-year-old children, irrespective of the experimental procedure. In Experiment 2, we explored how the size and number of cluster elements related to discomfort in 4- to 9-year-old children and adults through a remote experiment. The findings indicated that 4- and 5-year-olds exhibited increased discomfort as the number of cluster elements increased and the size of the elements decreased.

The results from Experiment 1 were in agreement with a previous face-to-face study^13^, in which 4- and 5-year-olds exhibited discomfort with trypophobic images. Additionally, Experiment 2 revealed that both children and adults experienced discomfort not only with natural images, such as lotus seed pods and beehives, but also with clusters of circles. This implies that visual attributes of circular object clusters elicit discomfort regardless of stimulus content. Furthermore, the cluster-size effect observed in children implies that the factors causing discomfort are similar in children and adults. In adults, other factors, such as the spatial structure of the image^1,7,14^ and fear of infectious diseases^2,3,5,14,17^, have been reported to influence discomfort. It is important to take these factors into consideration in future research.

In Experiment 1, a comparison of the ratings for trypophobic and neutral images revealed no differences according to age. This implies that, from around the age of 4 or 5 years, children experience the same level of discomfort as adults when exposed to trypophobic images, as previously reported by Suzuki et al. (2023)^13^. However, Experiment 2 revealed a more pronounced cluster-size effect in adults, particularly in relation to the disgust item. These results imply that discomfort with clusters may gradually increase from childhood to adulthood in some cases. It is evident that aversion to clusters is present from at least the age of 4 or 5 years, but the influence of cluster size and number changes with age. Furthermore, 4- and 5-year-olds tended to provide higher ratings for “fear” and “itchiness” in response to clusters compared to adults. This may have been because of preschoolers’ tendency to experience fear and anxiety in response to novel stimuli^18^.

While this study established that 4- and 5-year-olds experience discomfort with clusters, further research is needed to determine when this discomfort first emerges and how children develop avoidance responses to aggregates. It is possible that adaptive responses to avoid dangerous organisms may generalize to aggregates due to common features observed in images of toxic organisms and clusters^3^. However, studies investigating associations between trypophobia and toxic organisms through latent association tests with 4- and 5-year-olds have not provided evidence supporting this link^12^. If trypophobia has evolved as an adaptive response to avoid toxic organisms, it may manifest as early as infancy or even in other primate species, and it may be limited to circular clusters. Further research into the development of discomfort with clusters in young infants and non-human primates may enhance our understanding of the learning process and biological basis of trypophobia.

## Data Availability

The datasets generated and/or analyzed for the current study are available from the corresponding author on reasonable request.
